# Patient-Reported Financial Burden in Head and Neck Cancer Undergoing Radiotherapy

**DOI:** 10.3390/cancers18010003

**Published:** 2025-12-19

**Authors:** Renata Zahu, Monica Emilia Chirila, Otilia Ciobanu, Daniela Elena Sturzu, Andrei Ciobanu, Gabriela Ciobanu, Noemi Besenyodi, Madalina Vesel-Pop, Flavius Coșer, Roxana Costache, Gabriel Kacso

**Affiliations:** 1 Department of Oncology and Radiotherapy, “Iuliu Hațieganu” University of Medicine and Pharmacy, 400347 Cluj-Napoca, Romania; 2 Amethyst Radiotherapy Center Cluj, 407280 Florești, Romania; 3 Faculty of Medicine, Vrije Universiteit Amsterdam, 1081 HV Amsterdam, The Netherlands; 4 Amethyst Radiotherapy Center Timisoara, 307160 Timisoara, Romania; 5 Amethyst Radiotherapy Center Alba Iulia, 510040 Alba Iulia, Romania; 6 Amethyst Radiotherapy Center Bucuresti, 075100 Otopeni, Romania

**Keywords:** head and neck cancer, financial toxicity, financial burden, out-of-pocket payment

## Abstract

**Background/Objectives**: Financial toxicity (FT) refers to the financial burden directly or indirectly caused by a patient’s medical care. Patients with head and neck cancer (HNC) are particularly vulnerable to FT due to lower rates of return to work and higher out-of-pocket payments (OOPP). In this cross-sectional study, we assessed the amount and types of OOPP, as well as the prevalence of FT, in HNC patients who had completed curative radiotherapy. **Methods**: We included HNC patients who underwent curative-intent radiotherapy at four private clinics in Romania, within 12 months of completing treatment. Participants completed a 25-item questionnaire capturing sociodemographic information, insurance status, income, and OOPP. To assess subjective FT, we used the validated nine-item Financial Index of Toxicity (FIT), which measures three FT domains: financial stress, financial strain, and lost productivity. Each domain and the total score range from 0 to 100, with higher scores indicating greater financial toxicity. Descriptive statistics were used to summarize patient characteristics. Pearson’s chi-square, *t*-tests, and one-way ANOVA were used to assess statistical associations, with a significance threshold of *p* < 0.05. **Results**: Among 113 patients (mean age: 59), the majority were male (74.3%) and married (74.3%), with 40% having completed university or higher education. The most frequent tumor sites were the oropharynx (29 cases), larynx (22), and oral cavity (21). Concurrent chemoradiation was the most common treatment modality (47%). The mean total FT score was 18.8. Overall, 39.8% of patients experienced financial toxicity, and 29.2% scored above the mean in financial stress. Moderate financial strain (score > 21) was reported by 39.8% of participants, and approximately one-third reported loss of productivity. Transportation and nutritional supplements were the most common OOPP categories. Notably, 42% of patients spent at least 400 euros—equivalent to Romania’s monthly minimum income—on transportation during radiotherapy. FT was significantly associated with employment and marital status, but not with tumor site or treatment type. **Conclusions**: Among Romanian HNC patients treated with curative radiotherapy, we found substantial OOPP, particularly for transportation and nutritional supplements. While overall FT levels were moderate, divorced patients and those retired due to other chronic conditions were the most vulnerable to financial distress. Financial toxicity can directly affect treatment adherence, survival, and quality of life. By integrating financial counseling, social support, and broader coverage of treatment-related expenses, healthcare systems can mitigate FT for these patients.

## Simple Summary

Financial toxicity has been recently recognized as an important research topic and a prognostic factor for oncology patients’ outcomes. Head and neck cancer patients are at high risk for financial toxicity as they have lower work returns and more out-of-pocket costs due to treatment-related adverse effects. Real-world data about financial toxicity can help define the patients at risk and identify potential solutions to decrease its occurrence and impact. Our manuscript brings interesting data from an upper-middle-income country with a high prevalence of head and neck cancers and universal healthcare coverage. We included patients from four centers situated in different geographical areas, who received multidisciplinary treatment according to international guidelines, comprising standard-of-care radiotherapy. We evaluated both objective and subjective components of financial toxicity, including a validated questionnaire and the amount of out-of-pocket payments, and correlated the data with demographic and clinical parameters.

## 1. Introduction

Head and neck cancer (HNC) is the sixth most common malignancy in the world, with almost one million new cases per year [1]. Advanced-stage disease is treated by multimodal treatment including surgery, radiotherapy, and chemotherapy, which is associated with significant acute and late morbidity affecting eating habits, swallowing, hearing, speaking, physical appearance, and social behavior [2,3]. This leads to high direct and indirect healthcare costs [4]. During treatment, most patients lose their ability to work, and only 67% can successfully return to work. On a personal level, this is associated with loss of income and financial distress [5,6].

Financial toxicity (FT) is a term used to describe the financial burden directly or indirectly attributable to a patient’s medical care. This includes the cost of treatment-related complications but also non-medical costs, like housing and transportation. FT is associated with the decline of quality of life as well as increased mortality, possibly through avoidance to pursue additional treatment or non-compliance and lower survival [7,8,9,10].

Head and neck cancer (HNC) patients are at particularly high risk for FT as they have lower work returns, more out-of-pocket costs, and have an increased use of cost-coping strategies because of the numerous side effects caused by treatment [11,12]. Financial toxicity is significantly correlated with both decreased overall and cancer-specific survival and lower quality of life [10,13].

Financial toxicity varies among countries with different healthcare systems [14]. In a national public healthcare system with universal coverage, one would expect that patients should not experience cancer-related economic consequences, while in a mixed public–private or entirely private healthcare system, patients are more exposed to financial burden.

Massa and colleagues analyzed 16,771 US cancer patients and observed that head and neck cancer patients were not only more likely to be a racial or ethnic minority, to be poorer or less educated, and to have lower health status, but were also more likely to incur higher total healthcare costs and out-of-pocket medical costs [9]. In contrast, in a study from Norway with universal health coverage, no significant financial burden was found among HNC patients who underwent radiotherapy [15].

The aim of this study was to quantify the amount and types of out-of-pocket payments (OOPP), as well as the prevalence of FT, in HNC patients who had completed curative radiotherapy in a country with a high incidence of head and neck cancers.

## 2. Materials and Methods

The study used a cross-sectional design, employing a bottom-up approach to measure the prevalence of financial toxicity among Romanian head and neck cancer patients receiving radiotherapy. We included consecutive patients treated in four outpatient radiotherapy clinics. Between September 2024 and May 2025, patients were approached directly at the end of irradiation or during follow-up visits. Those who could not be contacted in person were invited by email or by phone. We included patients diagnosed with cancers of the nasopharynx, oropharynx, hypopharynx, larynx, oral cavity, or salivary glands who had completed radiotherapy or were within 12 months of completion. Exclusion criteria were the age less than 18, the presence of metastases at diagnosis, and the use of radiotherapy with palliative intent.

We collected the data using two sets of questions. The first set comprised 25 questions that collected data on tumor site, sex, ethnicity, marital status, educational level, income, living arrangements, socioeconomic status, income loss, out-of-pocket payments, and coping strategies. The estimated categories of out-of-pocket expenses included investigations not covered by the health insurance fund or the ones patients could not access in a timely manner, drugs and medications to cure treatment toxicity, complementary care with nutritional supplements, as well as travel and lodging to follow the treatment regimen. These expenses represent an objective financial burden. The questions also allowed free-text options to cover other less frequent situations.

The second set comprised the Financial Index of Toxicity (FIT) questionnaire, a validated instrument that measures subjective financial toxicity among head and neck cancer patients [16]. FIT incorporates a nine-item survey to measure three subdomains of FT: financial stress, financial strain, and lost productivity. Each item is graded on a 0–1 scale, and the total score is calculated as the mean of all response scores multiplied by 100; total scores range from 0 to 100, with higher scores indicating worse FT.

The questions that involved income or expenses allowed respondents to select an option without disclosing exact amounts. For the income questions, we used the Romanian currency and set thresholds based on Romanian incomes in 2024. The average pension for retired people was approximated as a minimum income (500 euros), and the average salary was approximated as the next threshold (1000 euros). The resulting categories were Low Income (below the minimum), Medium Income (between the minimum and the average), and High Income (above the average). For expenses, we estimated categories ranging from approximately 100 euros to more than 1000 euros. 

All analyses were performed in R version 4.3.1 (https://www.r-project.org/). Continuous variables were examined for distributional characteristics using histograms and Q-Q plots. Normality was formally evaluated using the Shapiro–Wilk. For comparison between two independent groups, an independent-samples *t*-test was used when both groups met the normality assumption. When normality was violated, the non-parametric Wilcoxon rank-sum test with continuity correction was applied instead.

For comparison across more than two independent groups, one-way ANOVA was performed when assumptions of normality and homogeneity of variances were satisfied. If these assumptions were not met, the non-parametric Kruskal–Wallis test was used. When the Kruskal–Wallis test indicated a statistically significant difference between groups, post hoc pairwise comparisons were conducted using Wilcoxon rank-sum tests with Bonferroni adjustment for multiple testing. The association between categorical variables was assessed using a chi-squared test of independence. A *p*-value < 0.05 was considered statistically significant.

## 3. Results

We invited 190 head and neck cancer patients (127 by email and 63 received a printed version). A total of 113 (59.4%) patients completed the questionnaires.

Within this cohort of 113 patients with a mean age of 59, ranging from 29 to 88, the majority were male (74.3%), married (74.3%), and 40% had a university or higher education level. National minorities like Hungarians, Ukrainians, and Moldavians represented 10%, and most patients come from urban areas. Most had national health insurance (98%) and were employed or retired. This means that the majority (86.6%) had a stable monthly income. Data is presented in Table 1.

The most common tumor location was the oropharynx, followed by the larynx and the oral cavity. The most common treatment modality was concurrent chemoradiation in 47% of respondents. Trimodal treatment with surgery and adjuvant chemoradiation was reported by 24.8%, and exclusive radiotherapy by 9.7% of the patients. Nearly half of the patients had radiotherapy at a distance from their hometown. Fifty patients (44%) needed to travel more than 100 km to obtain access. Most of the patients, 72.5%, had a monthly income of less than or equal to 1000 euros.

**Figure 1:**
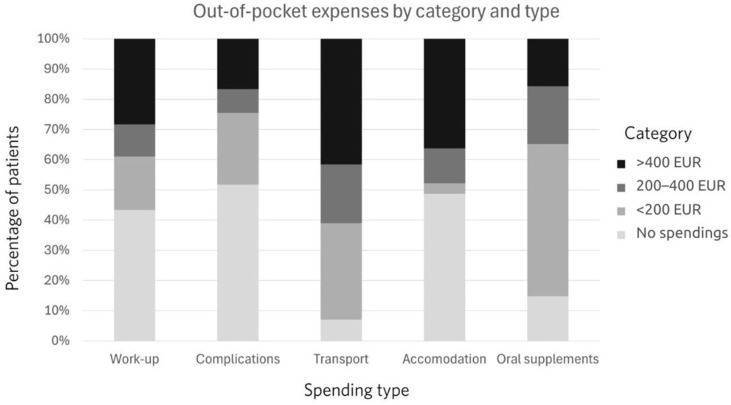
Out-of-pocket payments by amount and type.

Out-of-pocket payments were assessed for the whole course of treatment and are depicted in Figure 1.

**Figure 2:**
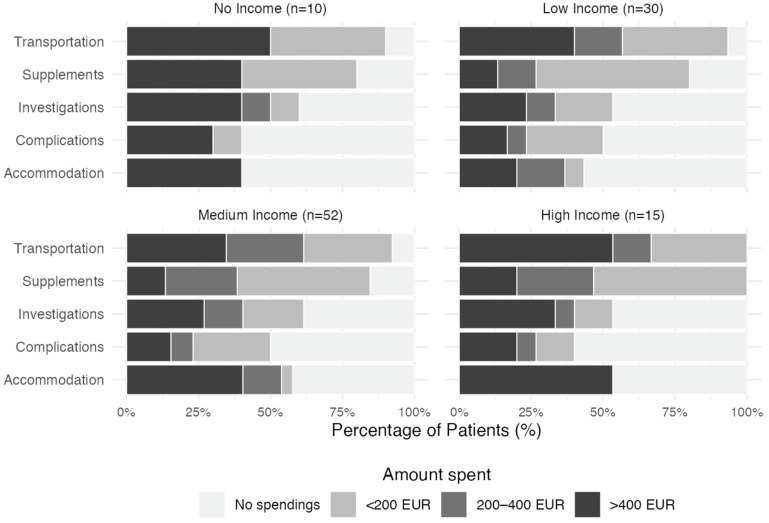
Proportional distribution of out-of-pocket payments across spending categories among patients who declared their monthly income. Income classifications are defined as: Low Income (<500 EUR), Medium Income (500–1000 EUR), and High Income (>1000 EUR).

The distribution of out-of-pocket spending differed significantly across spending types (χ^2^(12) = 140.09, *p* < 0.001). Sixty percent of patients spent at least 200 euros on transport. The second most frequent category for OOPPs was accommodation. Oral supplements represented the third most frequent category of spending. More than half, 51.3%, used up to 200 euros, 19.5% used from 200 to 400 euros, and 15.9% used more than 400 euros. Almost half of the patients had OOPPs for treating treatment-related complications. The income and OOPP relationship is depicted in Figure 2.

The mean total financial toxicity score was 18.8. More than a third of the patients (39.8%) presented financial toxicity, and 29.2% had higher than mean financial stress score. Forty-five patients (39,8%) reported financial strain scores over the mean of 21. A third of the patients reported loss of productivity.

On univariate analysis, younger age was significantly correlated with financial toxicity (*p* = 0.039), and this is mainly caused by loss of productivity, which was more prevalent in this age group. Being already retired for other chronic medical reasons at the time of the cancer diagnosis exposes patients to higher financial toxicity and financial strain (*p* = 0.015 and *p* = 0.007). Divorced patients reported significantly higher financial strain and stress scores than other patients (*p* = 0.011, *p* = 0.004). Gender, background, and education level were not statistically significant. Tumor-related parameters, such as tumor site and applied treatment combinations, did not have an impact on perceived financial toxicity. All correlations are presented in Table 2.

## 4. Discussion

In this study, we used a cross-sectional multi-institutional cohort of patients to examine patient-reported financial burden and out-of-pocket payments during curative radiotherapy for head and neck cancer. While tumor control remains the main objective in every treatment strategy, reducing acute and late side effects to improve survivors’ quality of life is also a high priority. Financial toxicity significantly impacts patients’ psychological and social wellbeing and has recently been recognized as one of the complications caused by cancer or its treatments [17,18,19]. Although FT does not manifest itself as a set of physical symptoms, clinicians should be aware of its importance, as several studies suggest it can influence both survival outcomes and treatment compliance [11,20]. Comprehensive clinical data is essential to fully appreciate its magnitude and develop strategies for mitigation.

In our cohort, the prevalence of self-reported financial burden was 39.8%. Ma et al. assessed financial toxicity in 284 patients with head and neck cancer treated with definitive or postoperative radiation therapy and found a prevalence of 14% associated with younger age, distant metastasis at diagnosis, HPV-negative tumor status, surgical treatment, and higher comorbidity burden. In that study, 17.9% of patients reported high levels of financial difficulty [10]. In our study, age was correlated with financial toxicity, but surgery or additional chemotherapy did not have any significant impact. This might be explained by the fact that we explicitly asked the patient about their perceived financial toxicity during radiotherapy.

Similarly, Rast et al. reported that 59.5% of 209 German patients with head and neck cancer experienced significant financial burden due to OOP payments and/or income loss [21].

In contrast, a Norwegian study of 67 head and neck cancer patients found only limited financial difficulty. The authors attributed this to Norway’s comprehensive social security system, which guarantees full salary compensation from the first day of work incapacity and provides access to additional compensatory payments such as spousal sick pay or welfare assistance [15].

Among American cancer patients with private health insurance, 42% reported significant or catastrophic financial burden. Of these, 68% reduced spending on leisure activities, 46% cut back on food and clothing, and 46% used personal savings to cover OOP expenses. To save money, 20% took less than the prescribed medication dose, 19% partially filled prescriptions, and 24% did not fill prescriptions at all [22]. Smith et al. observed that patients in the high-FT group were generally younger and more likely to be unmarried compared with those in the low-FT group [23]. This aligns with our findings that unmarried or divorced patients and those with chronic illnesses at the time of diagnosis were more likely to experience FT. In contrast, sex, education level, tumor location, and treatment type were not associated with higher financial hardship in our study.

As seen across studies, the degree of reported financial burden is heterogeneous and largely depends on each country’s social security and health insurance system.

The Romanian health insurance system is based on compulsory health and social insurance, providing full coverage for surgery, chemotherapy, and radiotherapy. However, patients still incur co-payments for certain prescription drugs, nutritional supplements, and some rehabilitation procedures. Employed patients can opt for paid sick leave and can apply for social assistance depending on their level of physical disability.

In our cohort, all patients reported some form of OOPP, most commonly for transportation, followed by nutritional supplements and accommodation. It is evident that 42% of the patients spent more than 400 euros on transportation during their radiotherapy treatment. Although the geographical distribution of radiotherapy facilities in Romania has improved in the last decade, with new private and public centers emerging in previously underserved areas, some patients still need to travel long distances or arrange accommodation near treatment centers. Massa et al. found that 38.9% of stress reported among head and neck cancer patients was attributable to medical expenses, while transportation concerns were the greatest source of stress for 27 patients. Combined surgery and adjuvant chemoradiation were reported as more stressful than surgery alone [9].

Interestingly, 32 of our patients reported OPPC exceeding 400 euros for diagnostic investigations (mostly CT and MRI) that were not available in a timely manner. Nutritional supplements are not covered by the national insurance system, yet 62% of patients reported spending up to 400 euros on them. Büttner et al. reported a mean monthly OOP expenditure of 206 euros during the first three months after hospitalization, decreasing to 148 euros after 15 months and an annual mean of 1776 euros [24]. Massa et al. reported a median annual OOP expenditure of 8384 dollars for head and neck cancer patients, significantly higher than for other cancer types [9]. However, cross-study comparisons remain challenging, as methodologies differ: some report raw OOP costs in different currencies, others report income proportions, and some categorize spending intervals.

Patients experiencing financial toxicity often adopt coping strategies, as described by Beeler et al. In their study, those with higher FT were more likely to reduce spending on food and clothing (54%), use savings (43%), or borrow money (13%) to cover treatment costs. These patients were also significantly more likely to refuse recommended tests, miss clinic visits, or not take prescribed medications [6]. Financial toxicity and the cost of care have a direct impact on treatment-related decisions. In a study by Fiala, efficacy accounted for 58% of all decisions, while the costs of treatment 22% on average [25].

To alleviate FT, both government and healthcare professionals can intervene through various measures. Paid sick leave remains the most important form of support, but additional programs—such as transportation and nutritional supplement reimbursements, as well as protocols facilitating survivors’ return to work—are also necessary [26]. Financial counseling provided by a dedicated full-time professional has proven effective in mitigating FT. Farrugia et al. reported that patients who did not receive financial counseling experienced significantly greater financial difficulty than those who did (*p* = 0.002) [27].

Our study has several limitations. Firstly, the participants in our study might not accurately represent the Romanian HN cancer patient population. Participation was voluntary and limited to outpatients, which may have led to underrepresentation of patients experiencing a higher financial burden. Additionally, inpatients might have reported lower financial distress and fewer OOP expenses. The high education level in the group of respondents might be interpreted as a selection bias. Patients with lower literacy might have been less prone to fill out the questionnaire. Thus, we might have underestimated the real level of FT in HN cancer patients, and caution is needed when extrapolating our results to patients with lower educational attainment.

Secondly, the heterogeneity of instruments used to measure FT further complicates cross-study comparisons. Some studies rely on a single question—such as item 28 from the EORTC QLQ-C30 questionnaire—which is insufficient to capture the complexity of FT and only reflects a short period of cancer experience. More comprehensive instruments include the Comprehensive Score for Financial Toxicity (COST), the Financial Distress Questionnaire (FDQ), the Subjective Financial Distress Questionnaire (SFDQ), the In Charge Financial Distress/Wellbeing Scale, the Socioeconomic Wellbeing Scale (SWBS), and the Patient-Reported Outcome for Fighting Financial Toxicity (PROFFIT) questionnaire [16,28,29,30,31,32].

We selected the Financial Index of Toxicity, as it is concise, easily understandable, and specifically validated in head and neck cancer patients. It captures the three key domains of FT: spending and resources, psychosocial effects, and coping behaviors. While our study was cross-sectional, a prospective design would allow for more precise evaluation of OOP costs throughout the cancer trajectory.

Thirdly, the level of FT may vary over time. We aimed to obtain a summary of experienced FT over a period of a maximum of one year after finishing radiotherapy. Since the interval from treatment completion was variable, some recall bias could have occurred in some patients.

With the continuous rise in treatment costs, understanding the financial burden of cancer survivors can help oncologists better counsel and support their patients during diagnosis and treatment. Multidisciplinary cancer care should incorporate rehabilitation services—such as physical and occupational therapy, speech therapy, financial counseling, and vocational rehabilitation—to help survivors return to work and reduce their financial burden.

## 5. Conclusions

Financial toxicity is a significant concern for patients with head and neck cancer, particularly among younger individuals with loss of productivity, those with pre-existing chronic medical conditions, and unmarried or divorced patients. Out-of-pocket expenses can reach substantial levels even within universal healthcare systems. Head and neck cancer patients are particularly vulnerable to treatment-related financial toxicity, which can directly affect treatment adherence, survival, and quality of life. Studying the impact of financial counseling, social support, and work reintegration strategies represents future areas to mitigate FT for these patients.

## Figures and Tables

**Table 1:** Pretreatment baseline parameters.

Clinical Characteristics (n = 113)	n (%)
Mean **age** (min, max)	59.3 (29.8)
**Sex**	
Male	84 (74.3)
Female	29 (25.7)
**Education level**	
High school or below	64 (56.6)
University or higher	44 (38.9)
Not disclosed	5 (4.4)
**Marital status**	
Married	84 (74.3)
Divorced	8 (7)
Widowed	8 (7)
Single	12 (10.6)
Not disclosed	1 (0.9)
**Area**	
Urban	69 (61)
Rural	44 (39)
**Employment status**	
Employee	41 (36.2)
Freelancer	5 (4.4)
Retired	57 (50.4)
Student	1 (0.9)
Social welfare	9 (8)
**Income level**	
No income	10 (8.8)
Low income	30 (26.5)
Medium income	52 (46)
High income	15 (13.2)
NA	6 (5.3)
**Tumor site**	
Nasopharynx	15 (13.3)
Oral Cavity	21 (18.6)
Oropharynx	29 (25.7)
Larynx	22 (19.5)
Hypopharynx	8 (7)
Nasal cavities and sinuses	3 (2.7)
Salivary gland	3 (2.7)
Not specified	12 (10.6)
**Treatment type**	
RT alone	11 (9.7)
Concomitant RT-CHT	53 (47)
Surgery + Adjuvant RT	21 (18.6)
Surgery + Adjuvant RT + CHT	28 (24.8)

**Table 2:** Univariate analysis of factors associated with financial toxicity.

	Financial Toxicity (Mean ± SD)	*p*	Financial Stress (Mean ± SD)	*p*	Financial Strain (Mean ± SD)	*p*	Loss of Productivity (Mean ± SD)	*p*
**Age**								
<65	20.2 ± 16.7	**0.039**	13.8 ± 19.4	0.405	23.3 ± 14.7	0.057	23.8 ± 34.0	0.087
>65	14.1 ± 11.7		11.0 ± 13.3		17.1 ± 14.1		13 ± 26.3	
**Gender**								
Female	17.2 ± 14.2	0.515	11.5± 17.2	0.553	19.8 ± 14.3	0.399	20.7 ± 28.4	0.909
Male	19.3 ± 16.4		13.7 ± 18.5		22.5 ± 14.9		21.4± 34.1	
**Background**								
Urban	19.6 ± 16.1	0.496	14.4 ± 19	0.321	22.7 ±14.9	0.393	21 ± 31.4	0.929
Rural	17.5 ± 15.5		11.06 ± 16.6		20.3 ± 14.5		21.6 ± 34.8	
**Education level**								
Lower secondary school	17.7 ± 14.8	0.413	12.2 ± 18.7	0.584	22.5 ± 14.3	0.392	16.1 ± 30.6	0.366
High school	16.7 ± 14.8		10.8 ± 14.4		19.9 ± 14.9		19.3 ± 30.9	
University	21.6 ± 17.2		16.3 ± 21.9		23.1 ± 14.8		26.4 ± 34.8	
Not disclosed	22.9 ± 21.2		15.5 ± 14.8		25 ± 17.7		30 ± 44.7	
**Tumor site**								
Nasopharynx	15 ± 14	0.357	11.7 ± 13.5	0.541	18.3 ± 14.3	0.241	13.3 ± 29.7	0.568
Oral Cavity	21.9 ± 16.8		15.3 ± 20.7		25.9 ± 14.6		23.8 ± 30.1	
Nasal cavity and sinuses	6.48 ± 1.6		0		14.6 ± 3.6		0	
Salivary glands	14.2 ± 6.9		3.67 ± 6.3		12.5 ± 6.3		33.3 ± 28.9	
Hypopharynx	22.2 ± 14.3		20.8 ± 21		28.1 ± 15.7		12.5 ± 23.1	
Larynx	22.6 ± 16.8		15.1 ± 18		24.7 ± 15.4		29.5 ± 39.8	
Oropharynx	14.9 ± 16.0		9.54 ± 17.0		17.7 ± 14.2		17.2 ± 33.5	
Other	22.4 ± 16.7		16.6 ± 22.5		23.4 ± 15.8		29.2 ± 33.4	
**Professional status**								
Social benefit	25.4 ±19	**0.015**	16.1 ± 25.6	0.131	25.7 ± 13.8	**0.007**	38.9 ± 41.7	0.102
Free lancer	20.5 ± 11		6.6 ± 6.02		26.2 ± 12.8		30 ± 44.7	
Retired due to another disease/illness	30.3 ± 18.1		25.3 ± 22.1		31.2 ± 15.1		35.7 ± 41.3	
Retired due to age	14.2 ± 13		12.1 ± 16.4		16.4 ± 13.1		12.8 ± 26.9	
Employee	18.4 ±15.8		10.3 ± 16.5		23.3 ± 14.7		20.7 ± 29.5	
Student	3.67 ± 0		11 ± 0		0		0	
**Marital status**								
Married	17.3 ± 16.2	0.06	11.6 ± 17.5	**0.011**	19.5 ± 13.6	**0.004**	21.4 ± 35	0.985
Divorced	33 ± 14.3		33.2 ± 21.5		36.7 ± 13.1		25 ± 26.7	
Widowed	18 ± 10.4		12.5 ± 17.3		21.9 ± 16		18.8 ± 25.9	
Unmarried	20.7 ± 14.3		10.1 ± 14.5		28.6 ± 16.7		20.8 ± 25.7	
Not disclosed	12.9 ± 0		22 ± 0		12.5		0	
**Treatment type**								
Surgery + RT	17.1 ± 14.1	0.878	6.33 ± 16	0.225	21.7 ± 12.5	0.995	23.8 ± 30.1	0.569
Surgery + RT + Chemo	19.6 ± 17.4		12.2 ± 16.7		22.3 ± 16.9		25 ± 34.7	
RT	16.4 ± 15.6		15.1 ± 18.7		21 ± 15.4		9.1 ± 30.2	
RT + Chemo	19.5 ± 15.9		15.9 ± 19.2		21.7 ± 14.6		20.8 ± 33.2	

Bold is for significant *p* values: *p* < 0.05.

## Data Availability

The datasets generated during the current study are available upon reasonable request from the corresponding author.

## Abbreviations

The following abbreviations are used in this manuscript:

HNC	Head and Neck Cancer
FT	Financial Toxicity
OOPP	Out-of-pocket-payments
FIT	Financial Index of Toxicity
CT	Computed Tomography
MRI	Magnetic Resonance Imaging
COST	Comprehensive Score for Financial Distress
FDQ	Financial Distress Questionnaire
SFDQ	Subjective Financial Distress Questionnaire
SWBS	Socioeconomic Wellbeing Scale
PROFFIT	Patient-Reported Outcome for Fighting Financial Toxicity
